# Evaluation of validity, reliability, and readability of AI chatbots for gestational diabetes mellitus: a multi-model comparative study

**DOI:** 10.3389/fpubh.2026.1760871

**Published:** 2026-02-04

**Authors:** Xinxin Wang, Shuyan Lin, Hui Liu, Chuanqing Li, Li Zhou, Rongkang Li

**Affiliations:** 1 Department of Obstetrics, Shenzhen Nanshan Maternity and Child Healthcare Hospital, Shenzhen, China; 2 Department of Urology, South China Hospital, Medical School, Shenzhen University, Shenzhen, China; 3 Department of Urology, Lanzhou University Second Hospital, Lanzhou University, Lanzhou, China

**Keywords:** artificial intelligence, gestational diabetes mellitus, large language models, patient education, readability

## Abstract

**Background:**

Gestational diabetes mellitus (GDM) is increasingly prevalent worldwide and is associated with substantial short- and long-term risks for mothers and offspring, making high-quality, accessible health information essential. At the same time, artificial intelligence (AI) chatbots based on large language models are being widely used for health queries, yet their accuracy, reliability and readability in the context of GDM remain unclear.

**Methods:**

We first evaluated six AI chatbots (ChatGPT-5, ChatGPT-4o, DeepSeek-V3.2, DeepSeek-R1, Gemini 2.5 Pro and Claude Sonnet 4.5) using 200 single-best-answer multiple-choice questions (MCQs) on GDM drawn from MedQA, MedMCQA and the Chinese National Medical Examination item bank, covering four domains: epidemiology and risk factors, clinical manifestations and diagnosis, maternal and neonatal outcomes, and management and treatment. Each item was posed three times to every model under a standardized prompting protocol, and accuracy was defined as the proportion of correctly answered questions. For public-facing information, we identified 15 core GDM education questions using Google Trends and expert review, and queried four chatbots (ChatGPT-5, DeepSeek-V3.2, Claude Sonnet 4.5 and Gemini 2.5 Pro). Two obstetricians independently assessed reliability using DISCERN, EQIP, GQS and JAMA benchmarks, and readability was quantified using ARI, CL, FKGL, FRES, GFI and SMOG indices.

**Results:**

Overall MCQ accuracy differed significantly across the six chatbots (*p* < 0.0001), with ChatGPT-5 achieving the highest mean accuracy (92.17%) and DeepSeek-V3.2 and Gemini 2.5 Pro performing comparably well, while ChatGPT-4o, DeepSeek-R1 and Claude Sonnet 4.5 scored lower. Newer model generations (ChatGPT-5 vs. ChatGPT-4o; DeepSeek-V3.2 vs. DeepSeek-R1) consistently outperformed their predecessors across all four domains. Among the four models evaluated on public-education questions, ChatGPT-5 achieved the highest reliability scores (DISCERN 42.53 ± 7.20; EQIP 71.67 ± 6.17), whereas Claude Sonnet 4.5, DeepSeek-V3.2 and Gemini 2.5 Pro scored lower. JAMA scores were uniformly low (0–0.07/4), reflecting poor transparency. All models produced text above the recommended sixth-grade reading level; ChatGPT-5 showed the most favorable readability profile (for example, FKGL 7.43 ± 2.42, FRES 62.47 ± 13.51) but still did not meet guideline targets.

**Conclusion:**

Contemporary AI chatbots can generate generally accurate and moderately reliable GDM-related information, with newer model generations showing clear gains in diagnostic validity. However, limited transparency and systematically high reading levels indicate that these tools are not yet suitable as stand-alone resources for GDM patient education and should be used as adjuncts to clinician counseling and professionally curated materials.

## 1 Introduction

Gestational diabetes mellitus (GDM), defined as glucose intolerance with onset or first recognition during pregnancy, is a growing global public health concern. Recent large-scale meta-analyses estimate a pooled global standardized prevalence of GDM was 14.0%, with substantial regional variation and rising trends over time, influenced by maternal age, obesity, and evolving diagnostic criteria, ranges from 7.1 to 10.4% in America to 7.8% in Europe, 14.6% in Africa and 20.8% in South-East Asia (1, 2). Even mild hyperglycaemia in pregnancy is associated with increased risks of preeclampsia, cesarean delivery, macrosomia, shoulder dystocia, neonatal hypoglycaemia, and long-term cardiometabolic disease in both mothers and offspring. In addition, GDM also increases the mother’s later-life risk of developing diabetes and cardiovascular disease, and elevates the risk of childhood obesity in her offspring, thereby underscoring the need for accurate diagnosis, effective management, and clear patient education around GDM (1–4).

Concurrently, the internet has become a major source of pregnancy-related health information. Systematic reviews show that most pregnant women use online resources to seek information about pregnancy, fetal development, and complications, often perceiving such information as useful and trustworthy (5, 6). Women with GDM, in particular, frequently rely on web-based materials and digital tools to support self-management and decision-making (7). However, the quality and readability of online information on GDM are inconsistent.

More recently, artificial intelligence (AI)-driven chatbots and large language models (LLMs), such as ChatGPT, DeepSeek, Gemini and other systems, have emerged as new, interactive sources of health information. These models can generate conversational, tailored answers to user questions, and are increasingly consulted for medical explanations and practical advice. Evaluations across several clinical topics have used tools such as DISCERN, Ensuring Quality Information for Patients (EQIP), the Global Quality Scale (GQS), and the Journal of the American Medical Association (JAMA) benchmarks to assess chatbot outputs, alongside standard readability indices (8–10). Overall, these studies report mixed but generally moderate reliability, incomplete transparency, and reading levels that commonly exceed recommended standards, highlighting both the promise and limitations of current AI chatbots in health communication.

Google Trends has become an established infodemiological tool for characterizing public interest and information-seeking behavior in health and disease (11). By analyzing aggregated search volumes for specific topics, it is possible to identify the questions that patients and the general public most frequently ask online, and to align educational content with real-world information needs. Against this background, the present study systematically evaluates four widely accessible AI chatbots, namely ChatGPT-5, DeepSeek-V3.2, Claude Sonnet 4.5 and Gemini 2.5 Pro, on GDM-related content. Specifically, this study aimed to assess the validity of these chatbots using standardized GDM multiple-choice questions, to examine the reliability of their answers to core public-education questions derived from Google Trends and expert input, and to quantify the readability of their responses relative to established benchmarks, while also comparing performance across different generations within the same model families.

## 2 Methods

### 2.1 Study design

**Figure 1:**
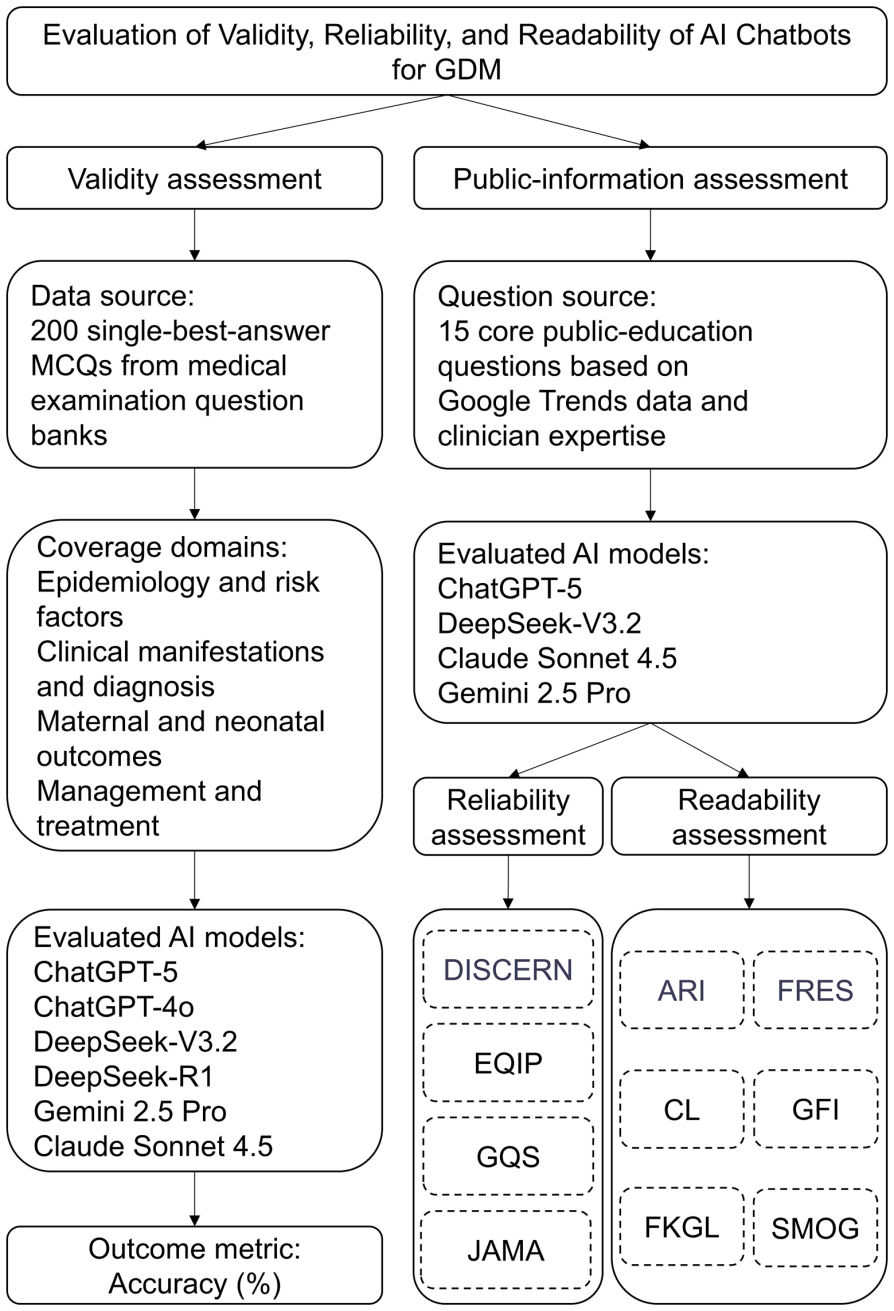
Workflow of study design and evaluation process. ChatGPT-4o and DeepSeek-R1 were included only in the validity analysis to assess iteration effects, whereas public-education analyses focused on the most recent model versions.

This study evaluates the responses of four artificial intelligence (AI) models, ChatGPT-5, DeepSeek-V3.2, Claude Sonnet 4.5, and Gemini-2.5 Pro, to standardized questions related to gestational diabetes mellitus (GDM), with a focus on their effectiveness, reliability, and readability. These models were selected based on their wide applicability, accessibility, and capacity to generate answers to medical and other health-related questions. We selected the evaluated LLM-based chatbots using predefined criteria: (i) public availability through official web interfaces without requiring developer-only access; (ii) broad real-world adoption for general information seeking, including health-related queries; (iii) representation of major model families from different developers to enhance cross-platform comparability; and (iv) active maintenance and recent version updates during the study period (data collection in November 2025). Based on these criteria, four widely accessible models were included for the public-education reliability and readability analyses (ChatGPT-5, DeepSeek-V3.2, Claude Sonnet 4.5, and Gemini 2.5 Pro). In addition, for the MCQ-based validity assessment, we included earlier generations within two model families (ChatGPT-4o and DeepSeek-R1) to evaluate the potential impact of model iteration on accuracy. The overall study workflow is schematically illustrated in Figure 1.

### 2.2 Data collection and analysis of validity

Experienced obstetricians curated a dataset of 200 single-best-answer multiple-choice questions (MCQs) on gestational diabetes mellitus (GDM), from two publicly available clinical question–answer datasets (MedQA and MedMCQA) and items from the Chinese National Medical Examination item bank (12, 13). The questions covered key domains related to GDM, including epidemiology and risk factors, clinical manifestations and diagnosis, maternal and neonatal outcomes, management and treatment. Each item had one pre-specified correct answer based on the original answer key. To avoid ambiguities related to translation, all questions were presented in their original language, and all models received identical wording and answer options.

For the validity assessment, each of the 200 MCQs was presented to six AI chatbots (ChatGPT-5, ChatGPT-4o, DeepSeek-V3.2, DeepSeek-R1, Gemini 2.5 Pro, and Claude Sonnet 4.5), under a standardized prompting protocol and in the same fixed order, without any additional contextual information, hyperlinks, figures, or images. For the MCQ-based validity assessment, all models were queried using the same standardized prompt: “Choose the single best answer (A, B, C, D, E) to the following question. Respond with only the letter of the correct option and nothing else. Question: [MCQ stem]. Options: A… B… C… D… E….” No additional contextual information or follow-up turns were provided. To account for random variability in model outputs, each model was queried three independent times for every item. Before each query, the conversation history was cleared to minimize potential carry-over and memory effects. The primary endpoint for validity was accuracy, defined as the proportion of items answered correctly out of the 200 questions for each model.

The study comprised two sets of analyses with distinct objectives. For the validity assessment, we included two generations within each model family (ChatGPT-4o and ChatGPT-5; DeepSeek-R1 and DeepSeek-V3.2) to examine the potential impact of model iteration on response accuracy. For the public-education quality assessment (DISCERN, EQIP, GQS, JAMA, and readability), we focused on comparing the most recent and representative versions across model families; thus, we included ChatGPT-5 and DeepSeek-V3.2 and excluded ChatGPT-4o and DeepSeek-R1 to reduce redundancy and to avoid interpreting quality metrics from earlier versions that were not the primary targets of the public-information comparison.

### 2.3 Core public-education questions on GDM

To ensure comprehensive coverage of terminology related to gestational diabetes mellitus (GDM), we first retrieved the MeSH heading “Diabetes Mellitus, Gestational” and its entry terms (Gestational Diabetes Mellitus, Diabetes, Pregnancy-Induced, Pregnancy-Induced Diabetes, etc.) and used these terms to query Google Trends. Google Trends automatically aggregated them under the unified topic “gestational diabetes mellitus,” which combines conceptually equivalent search terms across languages to reflect global search interest (11). We extracted the 15 most popular queries worldwide related to this topic over the past 5 years (2020–2025). Data were accessed in November 2025, and the exported query list is provided in Supplementary Table S1.

Several senior obstetricians reviewed these popular queries and, combined with their real-world clinical experience, refined them into 15 core, patient-oriented questions that reflect public information needs and clinical safety priorities (Table 1). Selection criteria included: (i) prominence in Google Trends; (ii) clinical importance and safety relevance; (iii) clarity for lay readers; and (iv) avoidance of redundancy. The final questions covered: (1) what GDM is (Q1); (2) who is at high risk (Q2); (3) common symptoms (Q3); (4) how the oral glucose tolerance test (OGTT) is performed during pregnancy (Q4); (5) how GDM is diagnosed (Q5); (6) whether GDM affects the course of pregnancy (Q6); (7) possible complications (Q7); (8) effects on the baby (Q8); (9) recommended blood glucose levels in daily life (Q9); (10) treatment options (Q10); (11) dietary considerations (Q11); (12) recommended exercise regimen (Q12); (13) whether treatment can fully restore normal blood glucose levels (Q13); (14) whether GDM affects the mode of delivery (Q14); and (15) whether women with GDM can breastfeed normally (Q15).

**Table 1:** Fifteen core public education questions on gestational diabetes mellitus.

Number	Question
1	What is gestational diabetes?
2	Who is at high risk for gestational diabetes?
3	What are the common symptoms of gestational diabetes?
4	How is the oral glucose tolerance test (OGTT) performed during pregnancy?
5	How is gestational diabetes diagnosed?
6	Does gestational diabetes affect the course of pregnancy?
7	What complications can gestational diabetes cause?
8	How does gestational diabetes affect the baby?
9	What blood glucose levels should be maintained in daily life for gestational diabetes?
10	What are the treatment options for gestational diabetes?
11	What dietary considerations should be followed for gestational diabetes?
12	What exercise regimen is recommended for gestational diabetes?
13	Can treatment for gestational diabetes fully restore normal blood glucose levels?
14	Will gestational diabetes affect the mode of delivery?
15	Can women with gestational diabetes breastfeed normally?

Each of the 15 questions was then posed verbatim to four AI chatbots (ChatGPT-5, Claude Sonnet 4.5, DeepSeek-V3.2, and Gemini 2.5 Pro), without additional instructions, role prompting, or supplemental context, and their full-text responses were collected for subsequent reliability and readability analyses. This minimal-prompt approach was chosen to approximate typical real-world patient information-seeking behavior. For the public-education analyses, each question was queried once per model to approximate a typical single-user interaction; response stability across repeated trials for these patient-facing questions was not assessed in the current study. Before each query session, browser data (cookies, cache, and history) were cleared to minimize potential bias from personalization or prior interactions.

### 2.4 Analysis of reliability

The reliability of chatbot-generated content was evaluated using four widely used tools: DISCERN, the Ensuring Quality Information for Patients (EQIP) instrument, the Global Quality Scale (GQS), and the Journal of the American Medical Association (JAMA) benchmark criteria.

DISCERN: DISCERN is designed to judge the quality of written information on health, particularly with regard to treatment choices (14). In line with previous studies, we adopted commonly applied score bands: 63–75 = excellent, 51–62 = good, 39–50 = fair, 27–38 = poor, and 16–26 = very poor quality (15).

EQIP: The EQIP tool is used to evaluate materials intended for patients, with attention to aspects such as clarity, completeness, and quality of writing (16). It includes 20 items, each rated as “yes,” “partly,” “no,” or “not applicable.” For scoring, responses are converted to numerical values (1 for “yes,” 0.5 for “partly,” and 0 for “no”). The total is divided by 20, and then the number of “not applicable” items is subtracted. This value is multiplied by 100 to obtain a percentage score (16). Following the original validation work, we reported the mean EQIP percentage for each response and categorized quality as follows: 76–100% = well written/excellent, 51–75% = good with minor shortcomings, 26–50% = notable quality problems, and 0–25% = severe quality deficiencies (17).

GQS: The GQS offers a global, user-centered judgment of online health information, taking into account overall usefulness, coherence and flow, and practicality for end users (18). It is based on a 5-point Likert scale, where 1 indicates very poor quality, 2 poor, 3 fair, 4 good, and 5 excellent.

JAMA benchmark criteria: The JAMA benchmarks assess whether health information meets four basic transparency criteria: identification of authors, appropriate attribution or references, indication of the date of last update, and disclosure of ownership or sponsorship (19). Each item is scored as 0 (absent) or 1 (present), yielding a total score ranging from 0 to 4.

All four tools (DISCERN, EQIP, GQS, and JAMA) were applied independently by two obstetricians with more than 10 years of clinical experience. Disagreements between the two primary raters were resolved through discussion to reach consensus. When consensus was not possible, a third senior obstetrician with approximately 20 years of experience acted as an arbitrator, and the adjudicated score was considered final.

Inter-rater reliability for the initial independent ratings (before consensus) was quantified. For total scores (DISCERN, EQIP, GQS, and JAMA), we computed the intraclass correlation coefficient using a two-way random-effects model with absolute agreement (ICC[A,1]) (20–22). For the four binary JAMA benchmark items, agreement was additionally assessed using Cohen’s kappa; when an item showed no variability across targets, kappa was not estimable and we reported the observed agreement. Inter-rater reliability of the initial ratings was high across instruments [DISCERN ICC = 0.956 (95% CI 0.917–0.980), EQIP ICC = 0.867 (95% CI 0.735–0.966), GQS ICC = 0.931 (95% CI 0.856–0.985), and JAMA ICC = 1.000 (95% CI 1.000–1.000)]. For JAMA items with no variability (all ratings were identical across targets), Cohen’s kappa was not estimable; however, the observed agreement was 100%.

### 2.5 Analysis of readability

We assessed the readability of the chatbot-generated answers using six commonly used readability metrics: the Automated Readability Index (ARI), Coleman–Liau Index (CL), Flesch–Kincaid Grade Level (FKGL), Flesch Reading Ease score (FRES), Gunning Fog Index (GFI), and SMOG index. All scores were calculated with an online readability tool^1^.

Among these indices, FRES is expressed on a 0–100 scale, where higher scores correspond to easier-to-read text. In contrast, FKGL, ARI, CL, GFI, and SMOG are reported as approximate U. S. school grade levels required to understand the content. Using this set of measures enabled us to compare the linguistic complexity of outputs across different chatbots and to evaluate how suitable their responses are for lay audiences seeking health information.

To contextualize readability, we aligned our evaluation with the sixth-grade reading level often recommended by the American Medical Association (AMA) and the National Institutes of Health (NIH). In accordance with these standards, we regarded a FRES score ≥ 80.0 as acceptable and required that each of the five grade-based indices (FKGL, ARI, CL, GFI, and SMOG) remain below 6 to be considered adequately accessible.

### 2.6 Statistical analysis

For each chatbot, answers to the 200 MCQs were coded as correct or incorrect, and item-level performance was summarized across three independent response attempts per question. Overall accuracy and error rates were then computed for each model. To facilitate comparison between chatbots, we expressed performance as the proportion (%) of correctly answered items. Differences in MCQ accuracy among models were examined with a one-way analysis of variance (ANOVA), adopting *p* < 0.05 as the threshold for statistical significance.

For the reliability metrics (DISCERN, EQIP, GQS, and JAMA) and readability indices (ARI, CL, FKGL, FRES, GFI, and SMOG), we calculated mean values and standard deviations for each chatbot. The Shapiro–Wilk test was applied to assess the normality of score distributions. Because many variables deviated from a normal distribution, we compared models on these outcomes using the nonparametric Kruskal–Wallis test.

To determine whether chatbot-generated texts satisfied the sixth-grade readability benchmark recommended by the AMA and NIH, we used the Wilcoxon signed-rank test to compare each readability index with its respective target (FRES ≥ 80.0, and <6 for each of the other five grade-level measures). Within each model, six hypothesis tests (one for each index) were conducted, and family-wise error rates were controlled using the Holm adjustment.

For inter-rater reliability, ICC(A,1) was calculated for each total score, and 95% confidence intervals were obtained using bootstrap resampling (5,000 iterations). All statistical procedures were carried out in R (version 4.5.0). Two-sided *p*-values < 0.05 were interpreted as statistically significant.

## 3 Results

### 3.1 Validity of AI chatbots on GDM multiple-choice questions

**Figure 2:**
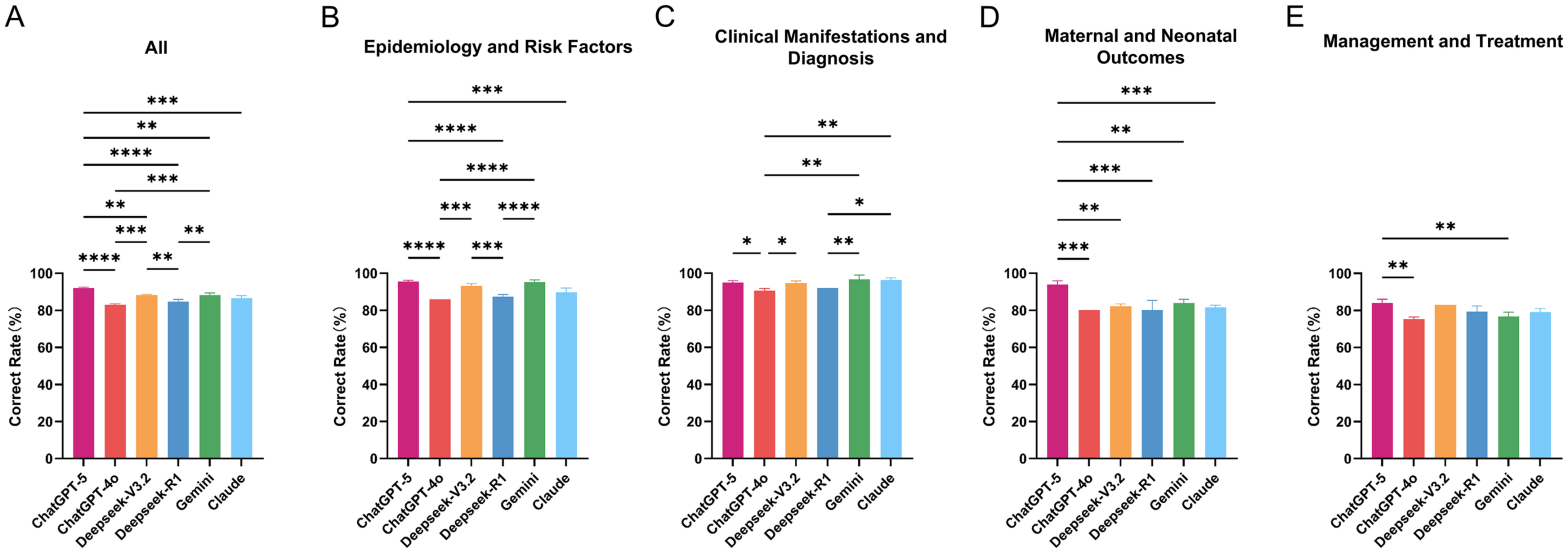
Validity of AI chatbots on gestational diabetes mellitus (GDM) multiple-choice questions. **(A)** Overall accuracy of six AI chatbots (ChatGPT-5, ChatGPT-4o, DeepSeek-V3.2, DeepSeek-R1, Gemini 2.5 Pro, and Claude Sonnet 4.5) on 200 single-best-answer multiple-choice questions (MCQs) related to GDM. **(B–E)** Domain-specific accuracy for the same six models across four content domains (50 MCQs each); **(B)** Epidemiology and risk factors; **(C)** Clinical manifestations and diagnosis; **(D)** Maternal and neonatal outcomes; and **(E)** Management and treatment. Bars represent the mean proportion of correctly answered questions (%) across three independent runs per item for each model; error bars indicate standard deviation. *p* values are from one-way ANOVA comparing accuracy between models within each panel, with significance levels denoted as *p < 0.05, **p < 0.01, ***p < 0.001, ****p < 0.0001.

Across the 200 GDM MCQs, overall accuracy differed significantly among the six chatbots (*p* < 0.0001; Figure 2A). Mean accuracy (± SD) was summarized in Table 2.

**Table 2:** Accuracy of AI chatbots in questions on gestational diabetes mellitus.

Category	ChatGPT-5	ChatGPT-4o	DeepSeek-V3.2	DeepSeek-R1	Gemini	Claude	*p*-value
All	92.17 ± 0.29	83.00 ± 0.50	88.33 ± 0.29	84.67 ± 1.26	88.17 ± 1.26	86.67 ± 1.26	<0.0001
Epidemiology and Risk Factors	95.67 ± 0.58	86.00 ± 0.00	93.33 ± 1.15	87.33 ± 1.15	95.33 ± 1.15	89.67 ± 2.31	<0.0001
Clinical Manifestations and Diagnosis	95.00 ± 1.00	90.67 ± 1.15	94.67 ± 1.15	92.00 ± 0.00	96.67 ± 2.31	96.33 ± 1.15	0.0006
Maternal and Neonatal Outcomes	94.00 ± 2.00	80.00 ± 0.00	82.33 ± 1.15	80.00 ± 5.29	84.00 ± 2.00	81.67 ± 1.15	0.0002
Management and Treatment	84.00 ± 2.00	75.33 ± 1.15	83.00 ± 0.00	79.33 ± 3.06	76.67 ± 2.31	79.00 ± 2.00	0.0011

Domain-stratified analyses showed similar heterogeneity (Figures 2B–E). For Epidemiology and Risk Factors, accuracy ranged from 86.00 ± 0.00% (ChatGPT-4o) to 95.67 ± 0.58% (ChatGPT-5) (*p* < 0.0001). For Clinical Manifestations and Diagnosis, all models performed well (90.67–96.67%), with a significant overall difference (*p* = 0.0006). In Maternal and Neonatal Outcomes, performance was more dispersed (80.00–94.00%), again with ChatGPT-5 at the top (94.00 ± 2.00%; *p* = 0.0002). For Management and Treatment, accuracies were lower overall (75.33–84.00%), but still varied significantly between models (*p* = 0.0011).

Within-model family comparisons highlighted the effect of product iteration. ChatGPT-5 outperformed ChatGPT-4o both overall (92.17% vs. 83.00%) and in every domain, with gains of 9.7, 4.3, 14.0, and 8.7 percentage points in Epidemiology and Risk Factors, Clinical Manifestations and Diagnosis, Maternal and Neonatal Outcomes, and Management and Treatment, respectively. Similarly, DeepSeek-V3.2 showed higher overall accuracy than DeepSeek-R1 (88.33% vs. 84.67%), with consistent, smaller advantages across all four domains. These patterns support that iterative updates of AI chatbots are associated with measurable improvements in the correctness of their answers to GDM-related MCQs.

### 3.2 Reliability analysis

**Figure 3:**
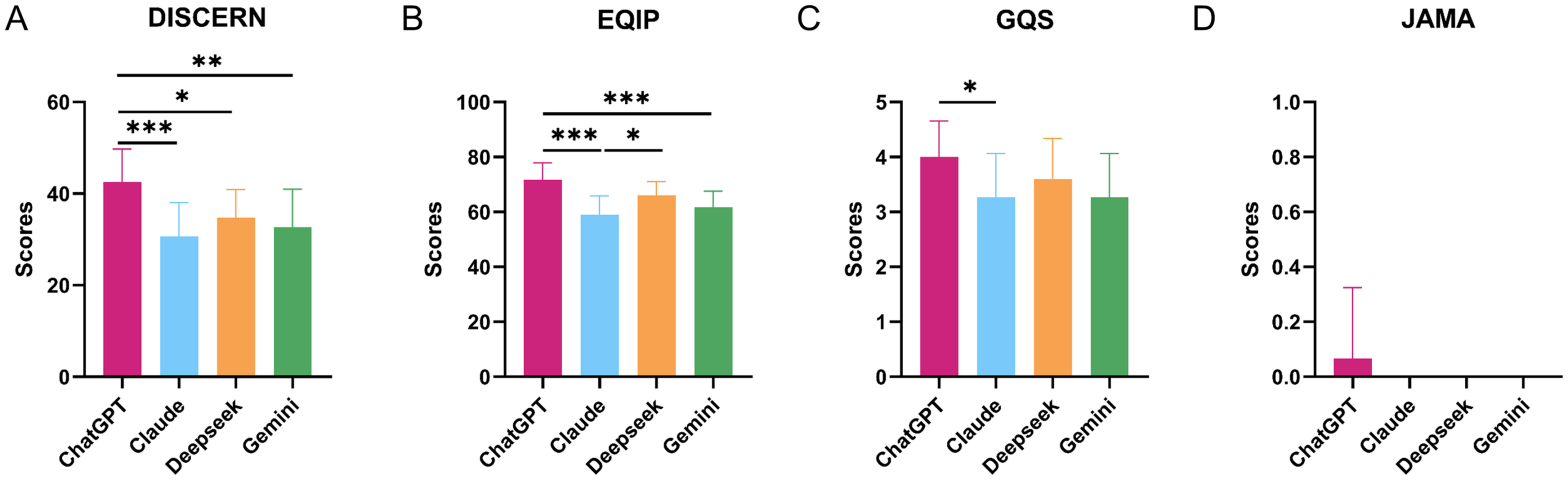
Reliability scores of AI chatbots for gestational diabetes mellitus (GDM) public-education questions. Bar plots show reliability scores for four AI chatbots (ChatGPT-5, Claude Sonnet 4.5, DeepSeek-V3.2 and Gemini 2.5 Pro) across four instruments: **(A)** DISCERN, **(B)** Ensuring Quality Information for Patients (EQIP), **(C)** Global Quality Scale (GQS), and **(D)** Journal of the American Medical Association (JAMA) benchmark criteria. Scores are calculated from responses to 15 core GDM public-education questions. Bars represent mean scores across the 15 questions for each chatbot; error bars indicate standard deviation. Overall *p*-values are derived from Kruskal–Wallis tests comparing the four chatbots for each instrument. Pairwise comparisons between chatbots use *post hoc* tests with adjustment for multiple comparisons, and statistically significant differences are indicated by asterisks: *p < 0.05, **p < 0.01, ***p < 0.001.

The reliability of responses from the four AI chatbots (ChatGPT-5, Claude Sonnet 4.5, DeepSeek-V3.2, and Gemini 2.5 Pro) is summarized in Table 3. Across instruments, ChatGPT-5 consistently achieved the highest mean scores. On DISCERN, ChatGPT-5 scored 42.53 ± 7.20, compared with 30.67 ± 7.37 for Claude Sonnet, 34.80 ± 6.10 for DeepSeek-V3.2, and 32.67 ± 8.30 for Gemini (overall *p* = 0.0011). Pairwise comparisons (Figure 3) showed that ChatGPT-5 performed significantly better than Claude Sonnet, DeepSeek-V3.2, and Gemini on DISCERN (adjusted *p* = 0.001, 0.0304, and 0.0065, respectively).

**Table 3:** Reliability scores across AI chatbots.

Program	DISCERN (mean ± SD)	EQIP (mean ± SD)	GQS (mean ± SD)	JAMA (mean ± SD)
ChatGPT	42.53 ± 7.20	71.67 ± 6.17	4.00 ± 0.65	0.07 ± 0.26
Claude Sonnet	30.67 ± 7.37	59.00 ± 6.87	3.27 ± 0.80	0 ± 0
DeepSeek	34.80 ± 6.10	66.00 ± 5.07	3.60 ± 0.74	0 ± 0
Gemini	32.67 ± 8.30	61.67 ± 5.88	3.27 ± 0.80	0 ± 0
*P*-value	0.0011	0	0.3916	0.0464

A similar pattern was observed for EQIP. ChatGPT-5 again had the highest score (71.67 ± 6.17), followed by DeepSeek-V3.2 (66.00 ± 5.07), Gemini (61.67 ± 5.88), and Claude Sonnet (59.00 ± 6.87), with a highly significant overall difference (*p* < 0.0001). *Post hoc* tests indicated that ChatGPT-5 scored significantly higher than Claude Sonnet and Gemini (adjusted *p* = 0 and 0.0006, respectively), and DeepSeek-V3.2 also exceeded Claude Sonnet (adjusted *p* = 0.0203).

For GQS, all models achieved relatively high mean ratings (3.27–4.00 on a 5-point scale), and the omnibus test was not significant (*p* = 0.3916). Nonetheless, pairwise analysis showed a small but statistically significant advantage for ChatGPT-5 over Claude Sonnet (adjusted *p* = 0.0449), while other pairwise differences were not significant. JAMA benchmark scores were uniformly low (0–0.07 out of 4), reflecting minimal reporting of authorship, references, or update dates by any chatbot. Although the overall comparison reached statistical significance (*p* = 0.0464), absolute differences between models were negligible.

**Figure 4:**
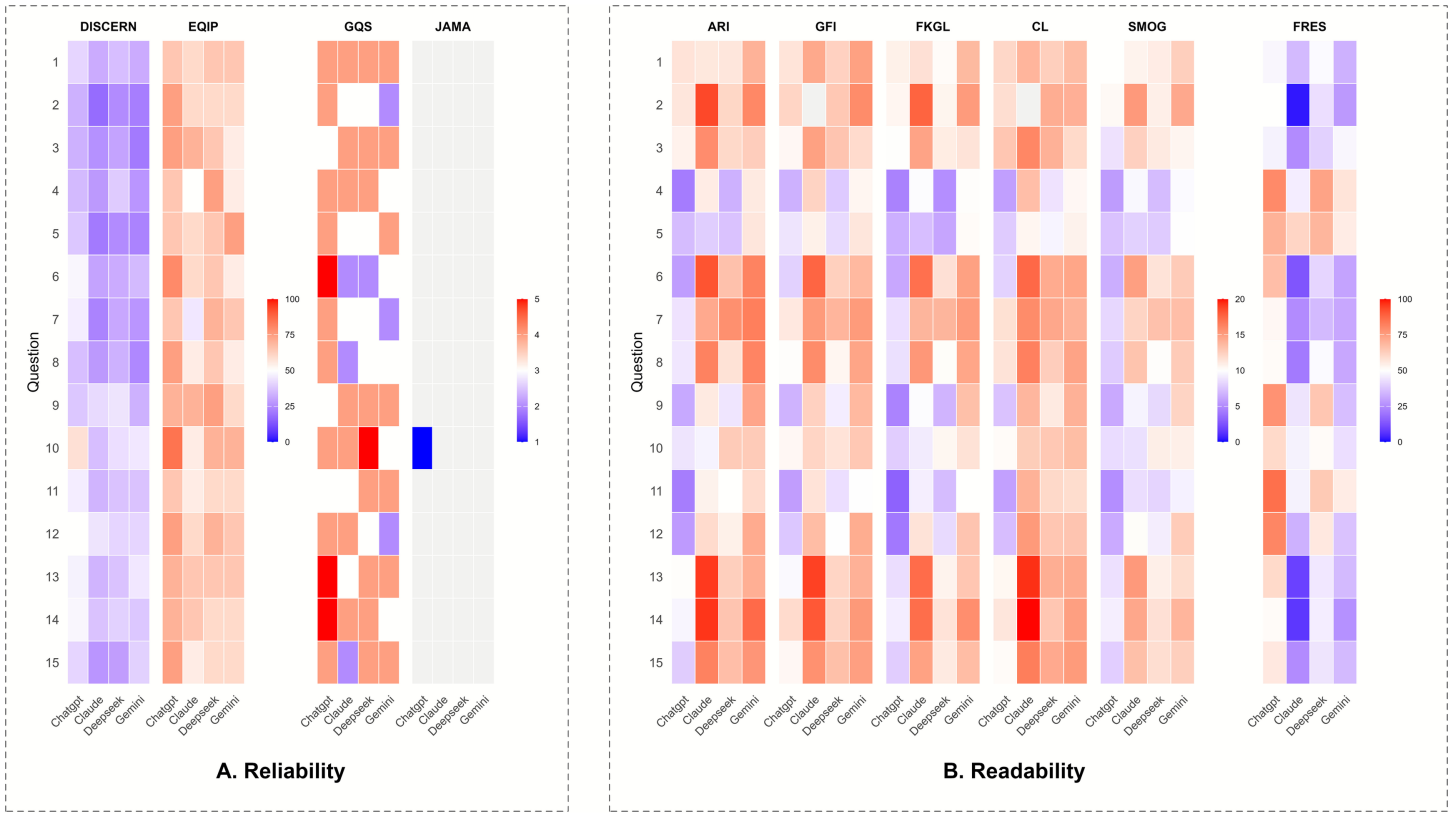
Item-level heatmaps for reliability and readability across the 15 core public-education questions. **(A)** Reliability. For each question (rows Q1–Q15), tiles display clinician-assigned scores for DISCERN, EQIP, GQS, and JAMA for four chatbots (*x*-axis order within each block: ChatGPT-5, Claude Sonnet 4.5, DeepSeek-V3.2, Gemini 2.5 Pro). Colors are mapped to the native range of each instrument as indicated by the side color bars; warmer colors correspond to higher scores. Ratings were performed independently by two obstetric clinicians, with discrepancies resolved by discussion and adjudication. **(B)** Readability. For the same questions, tiles show ARI, CL, FKGL, FRES, GFI, and SMOG computed from each chatbot’s answer (*x*-axis order as above). Warmer colors indicate higher numeric values. Note the interpretation: for ARI, CL, FKGL, GFI, and SMOG, higher values reflect a higher reading grade level and lower accessibility, whereas for FRES, higher values reflect easier text (0–100 scale). The heatmaps visualize per-item patterns that underlie the aggregate reliability and readability results presented in the bar plots.

Item-level reliability patterns across the 15 questions are depicted in the heatmap (Figure 4A). Scores varied by both question and model, with some items receiving consistently higher ratings and others showing weaker reliability across all chatbots. Visually, the heatmap corroborates the aggregate results from Table 3 and Figure 3, with ChatGPT-5 more frequently achieving higher DISCERN and EQIP ratings across individual questions, whereas Claude Sonnet, DeepSeek-V3.2, and Gemini display more variable and generally lower reliability profiles.

### 3.3 Readability analysis

**Figure 5:**
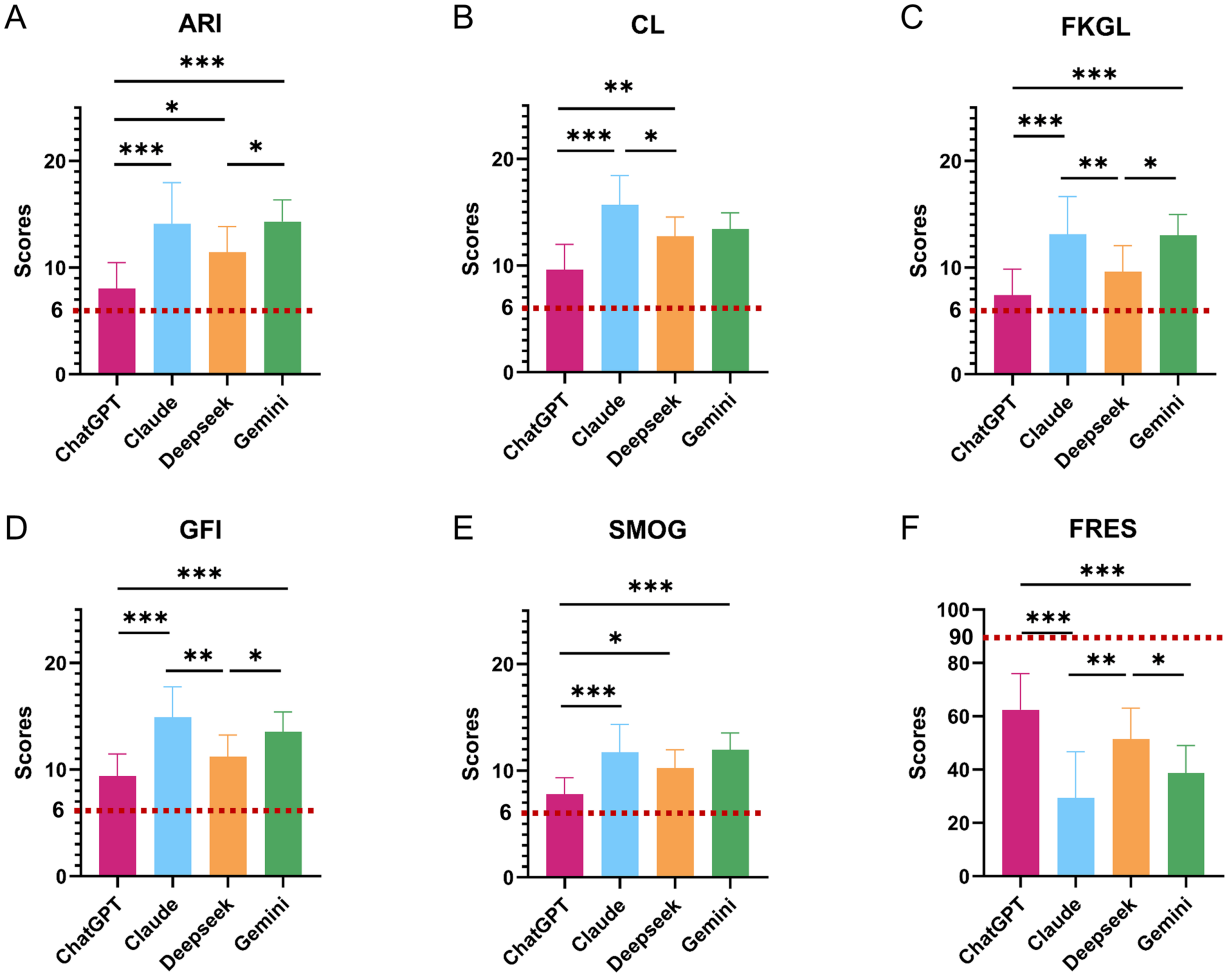
Readability scores of AI chatbots for gestational diabetes mellitus (GDM) public-education answers. Bar plots show readability scores for four AI chatbots (ChatGPT-5, Claude Sonnet 4.5, DeepSeek-V3.2, and Gemini 2.5 Pro) across six standard indices: **(A)** Automated Readability Index (ARI), **(B)** Coleman–Liau Index (CL), **(C)** Flesch–Kincaid Grade Level (FKGL), **(D)** Flesch Reading Ease score (FRES), **(E)** Gunning Fog Index (GFI), and **(F)** SMOG index. Scores are derived from responses to the 15 core GDM public-education questions. Bars represent mean values for each chatbot; error bars indicate standard deviation. For ARI, CL, FKGL, GFI, and SMOG, higher values indicate a higher estimated reading grade level and lower accessibility, whereas for FRES, higher values indicate easier text (0–100 scale). Overall *p*-values in each panel are from Kruskal–Wallis tests comparing the four chatbots. Pairwise differences are assessed with *post hoc* tests adjusted for multiple comparisons, with statistical significance denoted as *p < 0.05, **p < 0.01, ***p < 0.001.

Readability scores for the four chatbots are summarized in Table 4. Across all six indices, there were highly significant differences between models (all omnibus *p* < 0.0001; Figure 5). ChatGPT-5 consistently produced the most readable text, with lower grade-level estimates and higher FRES than the other systems (ARI 8.06 ± 2.42, CL 9.62 ± 2.37, FKGL 7.43 ± 2.42, GFI 9.41 ± 2.05, SMOG 7.78 ± 1.56; FRES 62.47 ± 13.51). In contrast, Claude Sonnet 4.5 and Gemini 2.5 Pro generated substantially more complex content (FKGL 13.11 ± 3.54 and 13.04 ± 1.96; FRES 29.33 ± 17.36 and 38.67 ± 10.39, respectively), while DeepSeek-V3.2 showed an intermediate profile (FKGL 9.64 ± 2.42; FRES 51.40 ± 11.65).

**Table 4:** Readability scores across AI chatbots.

Program	ARI (mean ± SD)	CL (mean ± SD)	FKGL (mean ± SD)	FRES (mean ± SD)	GFI (mean ± SD)	SMOG (mean ± SD)
ChatGPT-5	8.06 ± 2.42	9.62 ± 2.37	7.43 ± 2.42	62.47 ± 13.51	9.41 ± 2.05	7.78 ± 1.56
Claude Sonnet 4.5	14.11 ± 3.85	15.70 ± 2.76	13.11 ± 3.54	29.33 ± 17.36	14.91 ± 2.86	11.75 ± 2.60
DeepSeek -V3.2	11.43 ± 2.41	12.75 ± 1.80	9.64 ± 2.42	51.40 ± 11.65	11.23 ± 2.01	10.23 ± 1.73
Gemini 2.5 Pro	14.32 ± 2.02	13.42 ± 1.53	13.04 ± 1.96	38.67 ± 10.39	13.55 ± 1.86	11.97 ± 1.55
6th grade level score	6	6	6	80–90	6	6

Pairwise comparisons (Figure 5) confirmed that ChatGPT-5 was significantly easier to read than the other chatbots across most indices. ChatGPT-5 had lower ARI, FKGL, CL and SMOG scores than Claude Sonnet (all adjusted *p* ≤ 0.0001) and Gemini (all adjusted *p* ≤ 0.0007), and also lower ARI, CL and SMOG than DeepSeek-V3.2 (adjusted *p* = 0.0185, 0.0081 and 0.0149, respectively). FRES values showed the same pattern, with ChatGPT-5 scoring significantly higher than Claude Sonnet and Gemini (adjusted p = 0 and 0.0003, respectively), indicating easier-to-read output. Differences among the non-ChatGPT models were smaller but still evident, with Claude Sonnet generally yielding the highest grade levels and lowest FRES, and DeepSeek-V3.2 tending to be somewhat more readable than Claude Sonnet and Gemini. The item-level heatmap for readability (Figure 4B) illustrates similar patterns across individual questions, with ChatGPT-5 more frequently achieving the most favorable scores.

When benchmarked against the recommended sixth-grade readability targets (ARI/CL/FKGL/GFI/SMOG < 6 and FRES 80–90), none of the chatbots met the desired thresholds on any index. However, ChatGPT-5 consistently came closest to the target range, whereas the other three models typically produced material at a high-school to early college reading level.

## 4 Discussion

This study provides a comprehensive evaluation of four contemporary AI chatbots on GDM-related content, integrating measures of diagnostic validity, informational reliability, and textual readability. Overall, the models demonstrated high but heterogeneous accuracy on standardized MCQs, moderate to good reliability on structured quality instruments, and readability levels that generally exceeded recommended targets for patient-facing material. Taken together, these findings suggest that current AI chatbots can offer clinically reasonable information on GDM but should be used as adjuncts rather than stand-alone sources for patient education and decision-making.

In the context of the growing global burden of GDM and its well-established short- and long-term risks for mothers and offspring (1, 23, 24), our MCQ-based validity assessment demonstrated that all six models achieved relatively high overall accuracy, with ChatGPT-5 performing best on the full 200-item set and across all four clinical domains. DeepSeek-V3.2 and Gemini 2.5 Pro also achieved accuracy rates close to those of ChatGPT-5, whereas ChatGPT-4o, DeepSeek-R1 and Claude Sonnet 4.5 performed less well, particularly in questions related to maternal and neonatal outcomes and to management and treatment. These domain-specific patterns align with prior work suggesting that LLMs tend to perform better on factual recognition and diagnostic reasoning than on nuanced therapeutic decision-making or management trade-offs (8–10). Notably, within-model comparisons (ChatGPT-5 vs. ChatGPT-4o; DeepSeek-V3.2 vs. DeepSeek-R1) indicated that newer generations consistently outperformed their predecessors across all domains, supporting the notion that iterative model updates and training refinements translate into measurable gains in clinical answer accuracy.

The reliability analysis of responses to 15 core public-education questions, derived from Google Trends and expert review, showed that ChatGPT-5 delivered the most consistently reliable content, with mean DISCERN and EQIP scores in the “fair-to-good” range, whereas Claude Sonnet 4.5, DeepSeek-V3.2 and Gemini 2.5 Pro generally scored lower. These findings are broadly in line with earlier evaluations of ChatGPT and other models in various medical domains, which have reported moderate content quality and coherence when assessed with DISCERN, EQIP, GQS and related tools (8–10). Importantly, despite these relative differences, none of the chatbots approached the “excellent” range on DISCERN, and JAMA benchmark scores were near zero across all models, reflecting almost complete absence of explicit authorship, references, or update dates in the generated text. Similar deficits in transparency have been repeatedly documented in both traditional health websites and AI-generated content and raise important concerns regarding verifiability and accountability when chatbots are used for patient counseling or self-management support (25, 26). In parallel, studies evaluating the readability of online patient education materials across medical specialties have consistently reported similarly suboptimal readability, indicating that this limitation is not disease-specific but rather a broader challenge in both conventional and AI-assisted health information delivery (27–29).

The readability findings further refine the interpretation of these reliability results. Although ChatGPT-5 produced the most accessible text among the four models, with lower grade-level indices and higher FRES scores, none of the chatbots met the commonly recommended sixth-grade readability standard endorsed by the AMA and NIH. Instead, all models generated content that would generally require at least a late primary to high-school reading level, with Claude Sonnet 4.5 and Gemini 2.5 Pro frequently reaching early college levels. This mirrors prior analyses of both GDM-related websites and broader online health information, which consistently report reading levels well above recommended thresholds for the general public (25, 26). In parallel, studies evaluating the readability of online patient education materials across medical specialties have consistently reported similarly suboptimal readability, indicating that this limitation is not disease-specific but rather a broader challenge in both conventional and AI-assisted health information delivery. For example, postoperative patient instructions generated by ChatGPT have been compared with standard web search results in otolaryngology, with both sources frequently exceeding recommended reading levels (27). Likewise, systematic reviews in orthopedics have found that online patient education materials for total joint arthroplasty are commonly written above guideline targets (28). Similar concerns have also been reported in other reproductive and urologic contexts when AI chatbots are evaluated for patient-facing questions, including persistent elevation of readability grade levels (29). Our findings therefore suggest that current AI chatbots, despite their conversational style, may still produce text that is too complex for many patients with limited health literacy or numeracy, including those managing GDM. Given that women with GDM already report substantial challenges in locating, appraising and applying online health information (7, 30), this readability gap may limit the real-world impact of otherwise accurate and reasonably reliable chatbot outputs.

From a clinical and public health perspective, these results have several implications. First, the high MCQ accuracy—especially for newer model generations—suggests that AI chatbots could potentially assist clinicians with rapid access to guideline-consistent facts or be used as adjunct tools for medical education and exam preparation in obstetrics and endocrinology, provided that their outputs are critically appraised. Second, the moderate reliability but poor transparency highlight the ongoing need for human oversight when chatbots are used for patient counseling, particularly in high-stakes settings such as pregnancy and GDM. Third, the mismatch between chatbot readability and recommended literacy targets underscores the importance of integrating health-communication principles into LLM training and prompt design, for example by constraining responses to simpler language, shorter sentences and explicit numerical explanations. Finally, our use of Google Trends to derive core public questions illustrates how infodemiological data can help align AI evaluations with real-world information needs (30), and this approach could be extended to other pregnancy complications and chronic diseases.

This study has several strengths. It simultaneously evaluated validity (through 200 standardized MCQs), reliability (via four complementary quality instruments), and readability (across six indices), providing a multidimensional view of chatbot performance on a clinically important topic. The inclusion of multiple model families and generations, and the use of repeated queries with cleared histories, reduced random variability and allowed us to examine the impact of product iteration on performance. The derivation of public-education questions from both Google Trends and clinical expertise enhanced ecological validity by focusing on information that patients are likely to seek online.

Several limitations should also be acknowledged. First, this was a cross-sectional analysis of rapidly evolving AI systems; performance may change with subsequent updates, fine-tuning, or safety-layer modifications, limiting the temporal generalizability of our findings. Second, although the MCQ datasets and public questions covered key aspects of epidemiology, diagnosis, outcomes and management, they cannot fully capture the complexity of real-world clinical encounters, including nuanced shared decision-making, cultural factors and comorbidities. Third, reliability ratings based on DISCERN, EQIP, GQS and JAMA, while widely used, remain partly subjective, although we attempted to mitigate this through independent dual rating and adjudication. Fourth, we evaluated responses in a single language and did not test multimodal capabilities, so our findings may not extend to other languages or to image-based queries, for example, interpreting test reports or ultrasound images. Additionally, for the public-education questions, we analyzed a single response per model and did not quantify within-model response stability; future work should evaluate consistency of reliability and readability metrics across multiple independent trials. Finally, we did not assess actual patient comprehension, behavior change or clinical outcomes; future studies should investigate how chatbot-generated GDM information affects understanding, self-efficacy, glycaemic control and satisfaction among diverse patient populations.

In conclusion, contemporary AI chatbots can generate generally accurate, moderately reliable answers to GDM-related questions, with newer model generations showing clear gains in diagnostic validity. However, their outputs still lack basic transparency features and often exceed recommended readability thresholds, which may limit safe and equitable use for patient self-education. Until these limitations are addressed, AI chatbots should be viewed as promising but imperfect adjuncts to, not replacements for, evidence-based clinical counseling and professionally curated educational materials. Future work should focus on integrating explicit sourcing, version control and readability controls into chatbot design, as well as on co-developing and testing chatbot-assisted GDM education interventions with patients and clinicians.

## 5 Conclusion

In summary, this study showed that four widely accessible AI chatbots (ChatGPT-5, DeepSeek-V3.2, Claude Sonnet 4.5 and Gemini 2.5 Pro) can provide generally accurate answers to GDM-related multiple-choice questions, with newer generations outperforming earlier versions and ChatGPT-5 achieving the highest validity across all clinical domains. For core public-education questions, ChatGPT-5 also delivered comparatively more reliable information, although all models demonstrated limited transparency and produced text written above the recommended sixth-grade reading level. These findings suggest that current AI chatbots may serve as useful adjuncts for GDM information but are not yet suitable as stand-alone tools for patient education, underscoring the need for future model development to prioritize transparency, explicit sourcing and improved readability for diverse patient populations.

## Data Availability

The original contributions presented in the study are included in the article/Supplementary material, further inquiries can be directed to the corresponding authors.

## Footnotes

1
https://readabilityformulas.com/
