# Supplementary material for: Evaluation of validity, reliability, and readability of AI chatbots for gestational diabetes mellitus: a multi-model comparative study
Source: Front Public Health. 2026 Feb 4;14:1760871. doi: 10.3389/fpubh.2026.1760871 (PMC12913397; doi:10.3389/fpubh.2026.1760871)
Supplement: Supplementary file 1 [file Table_1.DOCX]

**Table S1** Google Trends data of the 25 most significant keywords queried globally for GDM between 2020–2025.

| TOP | Relevance |
| --- | --- |
| diabetes | 100 |
| gestational | 73 |
| gestational diabetes | 70 |
| diabetes gestacional | 22 |
| diabetes pregnancy | 15 |
| gdm | 14 |
| gestational diabetes pregnancy | 8 |
| 妊娠糖尿病 | 8 |
| gestational diabetes test | 7 |
| diabetes in pregnancy | 7 |
| tiểu đường thai kỳ, | 6 |
| schwangerschaftsdiabetes | 5 |
| diabete gestationnel | 5 |
| diabetes symptoms | 5 |
| what is gestational diabetes | 5 |
| سكر الحمل | 5 |
| gestational diabetes symptoms | 5 |
| diabete gestacional | 4 |
| gestational diabetes in pregnancy | 4 |
| estational diabetes baby | 4 |
| diabetes during pregnancy | 4 |
| diabetes mellitus | 3 |
| gestational diabetes diet | 3 |
| glucose test | 3 |
| diabète gestationnel | 3 |
